# From Leaves to Roots: Seasonal Dynamics of Wild Boar (*Sus scrofa*) Use of Understory Plant Resources in a Forest–Agricultural Landscape of Central Poland

**DOI:** 10.3390/ani16172688

**Published:** 2026-08-28

**Authors:** Paweł Nasiadka, Artur Obidziński, Magdalena Perlińska-Teresiak, Lidia Orłowska

**Affiliations:** 1 Faculty of Bioeconomy and Food Systems, Warsaw University of Life Sciences, Jana Ciszewskiego 8, 02-786 Warsaw, Poland; magdalena_perlinska_teresiak@sggw.edu.pl; 2 Faculty of Forestry and Wood Technology; Warsaw University of Life Sciences, Nowoursynowska 159, 02-776 Warsaw, Poland; artur_obidzinski@sggw.edu.pl; 3 The Institute of Biology and Earth Sciences, University of the National Education Commission, Krakow ul. Podchorążych 2, 30-084 Kraków, Poland; lidia.orlowska@uken.krakow.pl

**Keywords:** wild boar, food composition, omnivore, soil, rooting, understory vegetation

## Abstract

Wild boars (*Sus scrofa*) forage on plant resources located both above and below the soil surface, but it remains unclear whether these resources are exploited simultaneously or used as complementary food pools according to season. This study examined seasonal changes in the use of understory plant resources by analyzing the stomach contents of 470 wild boars collected throughout the year in central Poland. Four dietary fractions were quantified: grass leaves, herb leaves, grass roots, and herb roots. Their frequency and proportional contribution were compared among six phenological seasons using non-parametric statistics and generalized linear models. Significant seasonal differences were found in both occurrence and dietary contribution. Aboveground plant parts, particularly grass leaves, dominated during the Greening and Flowering periods, whereas underground organs became more important in autumn and winter. Herb roots showed the strongest seasonal increase during the Shedding season, while grass roots remained uncommon throughout the year. Our findings indicate that wild boars switch between these complementary food pools according to plant phenology, tracking seasonal changes in the availability and nutritional quality of plant tissues rather than exploiting aboveground and belowground resources indiscriminately. This flexible foraging strategy provides new insight into the ecological mechanisms underlying rooting behavior.

## Simple Summary

Wild boars are unique among European mammals because they feed on both aboveground vegetation and underground plant organs obtained by rooting. However, little is known about how the use of these food resources changes throughout the year. We analyzed the stomach contents of 470 wild boars collected in central Poland during six phenological seasons. The wild boars clearly adjusted their diet to seasonal plant development. Leaves dominated during spring and early summer, whereas underground plant organs became more important in autumn and winter. Grass leaves were the most important food resource throughout the year, while herb roots increased markedly at the end of the growing season. These findings provide further insight into seasonal variation in wild boar feeding ecology and the use of belowground plant resources through rooting.

## 1. Introduction

Wild boar (*Sus scrofa*) is among the most ecologically versatile large mammals in Europe [1,2,3]. Although traditionally classified as an omnivore, its diet under Central European conditions shows a clear predominance of plant-derived food. The diet of wild boar encompasses a broad spectrum of food resources, ranging from wild-growing herbaceous plants and tree seeds, through agricultural crops and meadow vegetation, to anthropogenic waste [1,4,5,6,7,8,9,10]. With landscape transformation and the increasing dominance of agricultural crops, the diet of wild boar, despite its omnivorous nature, is currently dominated by plant components [5,6,7,11,12].

Among large temperate-zone vertebrates, wild boars are distinguished by their ability to exploit food resources located within a narrow zone at and immediately below the soil surface. Whereas the consumption of aboveground parts of forest understory vegetation, as well as meadow and pasture vegetation, is typical of all ungulate species [13,14,15,16], wild boars are virtually the only species capable of rooting, i.e., actively digging through the upper soil layer in search of food [11,17,18,19]. Through rooting, they obtain small invertebrates and vertebrates [18,20,21] and underground plant parts including roots, rhizomes, tubers, bulbs, and seeds [6,7,22,23,24,25], as well as parts of cultivated plants [26,27,28].

The ecological effects of rooting have been the subject of numerous studies, focusing on its influence on soil structure [19,29,30] and on ecological processes within ecosystems, including the mixing of humus layers, fragmentation of dead organic matter [20,31,32], and facilitation of vegetative reproduction in certain plant species [33] but also soil desiccation and damage to seedlings and root systems [17,34,35,36].

Although the diet of wild boar has been the subject of numerous studies, comparatively little attention has been paid to how this species exploits food resources located both aboveground—in the form of aerial plant parts—and belowground—in the form of underground organs. It remains unclear whether these two resource types are exploited simultaneously or whether their contribution to the diet changes seasonally in response to variation in food availability. It is also unknown whether the narrow zone immediately above and below the soil surface constitutes a single foraging stratum that is exploited indiscriminately or whether wild boars distinguish between aboveground and belowground resources while foraging. These questions are of particular importance under the seasonally variable conditions of temperate ecosystems, where plant phenology, that is, the rhythmic pattern of plant development, including leaf emergence, Flowering, fruiting, and the autumn and winter senescence of aerial parts, plays a key role [37,38,39]. Phenological changes affect not only the quantity but also the quality of food available to herbivores, both above and below the soil surface. It can therefore be assumed that seasonal changes in the diet composition of wild boar reflect changes in both the availability and quality of particular types of plant resources.

In this study, particular attention was paid to comparing the contribution of grasses (Poaceae) and the remaining herbaceous understory plants, hereafter referred to as herbs. This distinction reflects differences in the ecology and availability of these two plant groups. Owing to their widespread occurrence and persistence—particularly in anthropogenic and agricultural habitats frequently exploited by wild boar, such as meadows and pastures [5,40,41]—grasses represent a relatively stable food resource. In contrast, herbaceous plants other than grasses constitute a more dynamic component of the vegetation, subject to seasonal and spatial variation and characteristic of forest understory and fallow land [42]. However, it remains unclear whether these two groups differ significantly in their contribution to the diet of wild boar and which of their parts (aboveground or underground) are preferentially exploited during different seasons.

The aim of the present study was to assess seasonal variation in the use of aboveground and underground parts of understory plants by wild boars and to compare the contribution of the two major vegetation groups—grasses and herbs—to their diet composition. In particular, the following research questions were addressed: Do wild boars exploit aboveground and underground parts of understory plants in similar proportions throughout the year, across different seasons? Are the observed differences driven by seasonal variation and plant phenological cycles? Do grasses and herbs differ in their contribution to the diet of wild boars and in the extent to which their aboveground and underground parts are exploited?

## 2. Materials and Methods

### 2.1. Study Area

**Figure 1:**
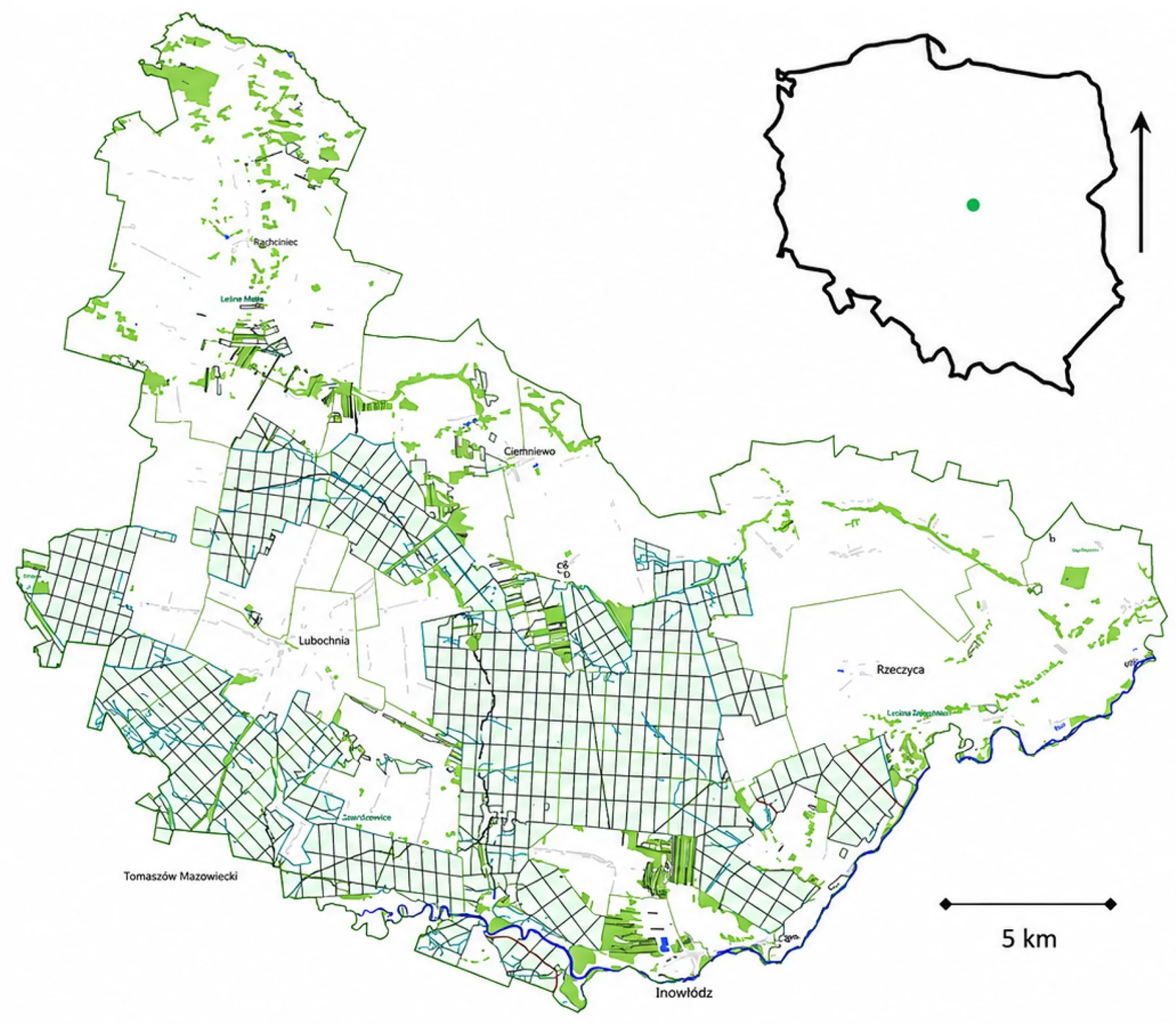
Location of forests (green) and agricultural land (white) within the Spała Forest District, central Poland.

This study was conducted in the Spała Forest District (51°32′ N, 20°08′ E), located in central Poland within the Pilica Primeval Forest (Puszcza Pilicka). The research area covered 15,585 ha, including 10,159 ha of forest and 5426 ha of agricultural land, forming a typical forest–agricultural mosaic landscape characteristic of central Poland (Figure 1).

The forested part of the study area consists of a large and relatively compact forest complex shaped by long-term forest management. Scots pine (*Pinus sylvestris*) is the dominant tree species and occupies more than 70% of the forest area. Despite the predominance of pine stands, relatively fertile forest habitats prevail, particularly fresh mixed coniferous forests and fresh mixed deciduous forests. The most common plant communities include *Leucobryo-Pinetum* in coniferous stands and *Tilio-Carpinetum* in deciduous forests. A characteristic feature of both habitat types is a well-developed understory vegetation layer, providing abundant cover and food resources for large herbivores and omnivores.

The forest complex is surrounded by a heterogeneous agricultural landscape dominated by small private farms. Cereal crops constitute the principal land use and occupy more than 60% of the agricultural area. Meadows and pastures account for approximately 15%, while potato fields, maize, legume crops and other agricultural land uses account for the remaining approximately 25%. The region is sparsely urbanized and lacks major urban centers.

Wild boars are among the dominant ungulate species inhabiting the study area. According to the State Forest Service, the local wild boar population is estimated at approximately 400 individuals (2.5 individuals km^−2^) based on year-round direct observations of wildlife conducted by State Forest Service personnel during the preceding year. Other ungulate species occurring in the area include red deer (*Cervus elaphus*), roe deer (*Capreolus capreolus*), and fallow deer (*Dama dama*). The combination of extensive forest cover, a well-developed understory, and the close proximity of agricultural habitats create favorable conditions for year-round wild boar foraging.

### 2.2. Data Collection and Sample Preparation

A total of 470 wild boars were harvested during regular individual hunts conducted throughout the year within the study area. Hunting was carried out from high seats located both within forest habitats and in the surrounding agricultural landscape. Only animals harvested during evening and early night hours were included in this study. All sampled individuals were harvested as part of routine wildlife management activities. Given the predominantly nocturnal foraging activity of wild boar in Central European landscapes, sampling during this period was intended to capture recently ingested food. Immediately after harvest, intact stomachs were removed from carcasses and transported for further processing. Only undamaged stomachs were used in the analyses. To minimize post-mortem digestion and decomposition, stomachs were frozen within 45 min of collection.

The study period was divided into six phenological seasons to account for seasonal changes in vegetation development and associated changes in the availability of plant resources. The classification and approximate duration of the phenological periods were based on botanical descriptions of plant development and on phenological classifications previously proposed for Poland. In particular, the classification applied in this study was a modification of the divisions proposed by Sokołowska (1965, 1980) [43,44] and the phenological system used in Poland since 2007 by the Institute of Meteorology and Water Management—National Research Institute [45]. The beginning and end of phenological periods are not fixed calendar dates and may vary between years depending on environmental conditions. Such variability has been documented, among others, by Siłuch and Bartoszek (2012) [46] and Siłuch et al. (2016) [47], who demonstrated a strong relationship between vegetation phenology and meteorological and remote-sensing observations. Therefore, the phenological periods used in the present study were treated as time intervals defined by vegetation development rather than as fixed calendar periods.

The six periods were Dormant (December and January), a period of vegetative dormancy, with little or no active aboveground plant growth and a predominance of Dormant or senescent plant material [48]; Reawakening (February and March), a period marking the onset of vegetation activity, characterized by the resumption of plant growth and the initial development of new shoots and leaves; Greening (April and May), a period of intensive vegetative growth, characterized by rapid development of leaves and aboveground biomass. In temperate grasslands, spring is generally associated with the highest rates of vegetation growth, preceding or accompanying the onset of reproductive development [48]; Flowering (June and July), a period of intensive reproductive development, characterized by Flowering of grasses and herbaceous plants and continued accumulation of aboveground biomass. The transition from vegetative to reproductive growth is also associated with changes in plant nutritional quality [49]; Ripening (August and September), a period of fruit and seed development and maturation, accompanied by progressive maturation and senescence of aboveground tissues. As plants mature, structural fiber generally increases, whereas crude protein concentration and digestibility tend to decline [50]; and Shedding (October and November), a period of progressive senescence, discoloration, and Shedding of leaves and aboveground plant parts, with a decline in active green biomass and an increasing contribution of senescent plant material.

In the laboratory, stomachs were thawed separately and their contents thoroughly mixed prior to subsampling. Mixing was performed to increase the likelihood that the collected material represented food consumed throughout the entire foraging period, including material located near the pyloric region as well as recently ingested food present in the cardiac portion of the stomach. From each stomach, a 200 mL subsample was collected for further processing.

The stomach contents were washed under running water through a set of sieves with mesh sizes of 3, 2, and 1 mm. Material retained on the 1 mm and 3 mm sieves was processed separately and used for other dietary analyses not considered in the present study. From the material retained on the 2 mm sieve, a 25 mL subsample was collected.

Each subsample was manually separated into dietary fractions. Four fractions representing aboveground and belowground components of understory vegetation were subsequently identified and isolated: grass leaves (GLs), herb leaves (HLs), grass roots (GRs), and herb roots (HRs). Additional dietary fractions present in the sample were also separated but were not considered in the present study.

All fractions obtained from each 25 mL subsample were dried at 75 °C for 24 h and weighed to the nearest 0.001 g. The combined dry mass of all fractions represented the total dry mass of the subsample. The dry masses of GLs, HLs, GRs, and HRs were then expressed as proportions of the total dry mass of the corresponding 25 mL subsample. These proportions formed the basis for all subsequent analyses of seasonal variation in understory resource use by wild boar.

### 2.3. Data Analysis

All statistical analyses were performed in R version 4.4.3 [51] using the tidyverse [52], rstatix [53], vegan [54], and ggplot2 [55] packages. The significance level was set at α = 0.05. For multiple comparisons, *p*-values were adjusted using the Holm method.

#### 2.3.1. Response Variables and Data Structure

For each stomach sample, the dry mass of the four understory fractions (GRs, GLs, HRs, HLs) was expressed as a proportion of the total dry mass of the 25 mL subsample. Two complementary response variables were derived for each fraction:

Frequency (presence), a binary variable indicating whether a given fraction was recorded in the sample (1 = present, 0 = absent), defined as a proportion greater than zero.

Proportional contribution, the continuous proportion of total dry mass contributed by each fraction.

Samples were grouped into six phenological periods: Dormant, Reawakening, Greening, Flowering, Ripening, and Shedding. In addition, fractions were aggregated into two higher-level categories: grasses (GRs + GLs) versus forbs (HRs + HLs), and belowground parts (GRs + HRs) versus aboveground parts (GLs + HLs). The combined proportional contribution of all four fractions within a sample (hereafter referred to as total understory contribution) was also calculated.

Because proportional data were strongly zero-inflated and right-skewed, non-parametric methods were used throughout. Descriptive statistics are reported as means, medians, and standard deviations (SDs).

#### 2.3.2. Seasonal Variation in Frequency

To test whether the frequency of occurrence of each fraction differed among phenological periods, contingency tables (season × presence) were analyzed separately for GRs, GLs, HRs, and HLs using Pearson’s chi-squared tests. When expected cell counts were too low, Fisher’s exact test was applied instead.

#### 2.3.3. Comparison of the Four Fractions Across the Entire Dataset

To compare the proportional contributions of GRs, GLs, HRs, and HLs across all samples, a Friedman test was used. This test accounts for the dependent structure of the data, as all four fractions were measured within the same stomach. When the global test was significant, pairwise differences were assessed with Wilcoxon signed-rank tests for paired samples, with Holm-adjusted *p*-values.

#### 2.3.4. Seasonal Variation in Proportional Contribution

For each fraction separately, differences in proportional contribution among phenological periods were tested with the Kruskal–Wallis test. Significant global effects were followed by Dunn’s post hoc tests with Holm correction.

Conversely, within each phenological period, differences in proportional contribution among the four fractions were tested with the Kruskal–Wallis test, followed by Dunn’s post hoc tests where appropriate.

The seasonal dynamics of total understory contribution (sum of all four fractions) were analyzed using the Kruskal–Wallis test, with Dunn’s post hoc comparisons applied when the global test was significant.

#### 2.3.5. Grouped Comparisons: Grasses vs. Forbs and Roots vs. Leaves

Differences in proportional contribution between grasses and forbs, and between belowground and aboveground parts, were tested with the Mann–Whitney U test (Wilcoxon rank-sum test). These comparisons were performed both for the entire dataset and separately within each phenological period.

#### 2.3.6. Logistic Regression of Fraction Presence

To assess the effects of phenological period, food fraction, and their interaction on the probability of occurrence of each fraction, a generalized linear model (GLM) with binomial error distribution and logit link was fitted:

presence~season × fraction

The Dormant season and grass roots (GRs) were used as reference categories. Model coefficients were exponentiated and reported as odds ratios (ORs) with 95% confidence intervals (CIs). Results were visualized in a forest plot on a logarithmic scale.

#### 2.3.7. Relationship Between Frequency and Proportional Contribution

For each fraction, the association between mean seasonal frequency and mean seasonal proportional contribution was assessed using Spearman’s rank correlation coefficient. This analysis was based on six paired observations (one per phenological period) for each fraction.

#### 2.3.8. Diet Diversity and Seasonal Similarity

Dietary diversity was quantified using the Shannon–Wiener diversity index (H′):

H′ = −Σ p_i_ ln(p_i_)

where *p*_i_ is the proportional contribution of fraction *i*. Two complementary approaches were applied:

Individual-level diversity: H′ was calculated for each stomach sample based on the four fractions, and mean values were computed for each phenological period.

Population-level diversity: H′ was calculated from mean seasonal proportions of the four fractions, reflecting the diversity of the average seasonal diet composition.

Similarity in diet composition between phenological periods was assessed using Schoener’s diet overlap index (*C*):

*C* = 1 − ½ Σ |*p*_ij_ − *p*_ik_|

where *p*_ij_ and *p*_ik_ are the mean proportional contributions of fraction *i* in seasons *j* and *k*, respectively.

Values range from 0 (no overlap) to 1 (identical composition). Pairwise Schoener indices were calculated from mean seasonal proportion matrices and visualized as a heatmap. Hierarchical clustering (UPGMA, average linkage) was applied to the dissimilarity matrix (1 − *C*) to explore groupings of phenological periods with similar dietary structure.

## 3. Results

The collected material comprised 470 stomach samples obtained across six seasonal periods: 80 (17.0%) in the Dormant season, 64 (13.6%) in Reawakening, 79 (16.8%) in Greening, 66 (14.0%) in Flowering, 53 (11.3%) in Ripening, and 128 (27.2%) in Shedding. Fractions of GRs, GLs, HRs, and HLs were recorded in 82.5% of all samples. Their frequency exceeded 90% during Flowering (97.0%), Greening (94.9%), and Ripening (90.6%). Lower occurrence was noted in Reawakening (79.7%) and Shedding (77.3%), while the lowest frequency of both aerial and underground herbaceous plant parts was observed in the Dormant season, where these fractions were found in only 63.8% of samples. Fractions of GRs, GLs, HRs, and HLs occurred in various configurations and frequencies. Across all seasons, the presence of one (*n* = 157; 33.4%) or two fractions (*n* = 174; 37.0%) was most common. Three fractions were detected in 52 cases (11.1%), while all four fractions occurred simultaneously only sporadically (*n* = 5; 1.1%). In samples containing two or three fractions, two combinations were particularly dominant. For three-fraction cases, the HRs × GLs × HLs configuration prevailed (*n* = 41; 78.8%), whereas among samples with two fractions, GLs × HLs was the most frequent (*n* = 129; 74.1%). Of the samples containing a single fraction, GLs were the most common (*n* = 81; 51.6%) (Table 1).

**Figure 2:**
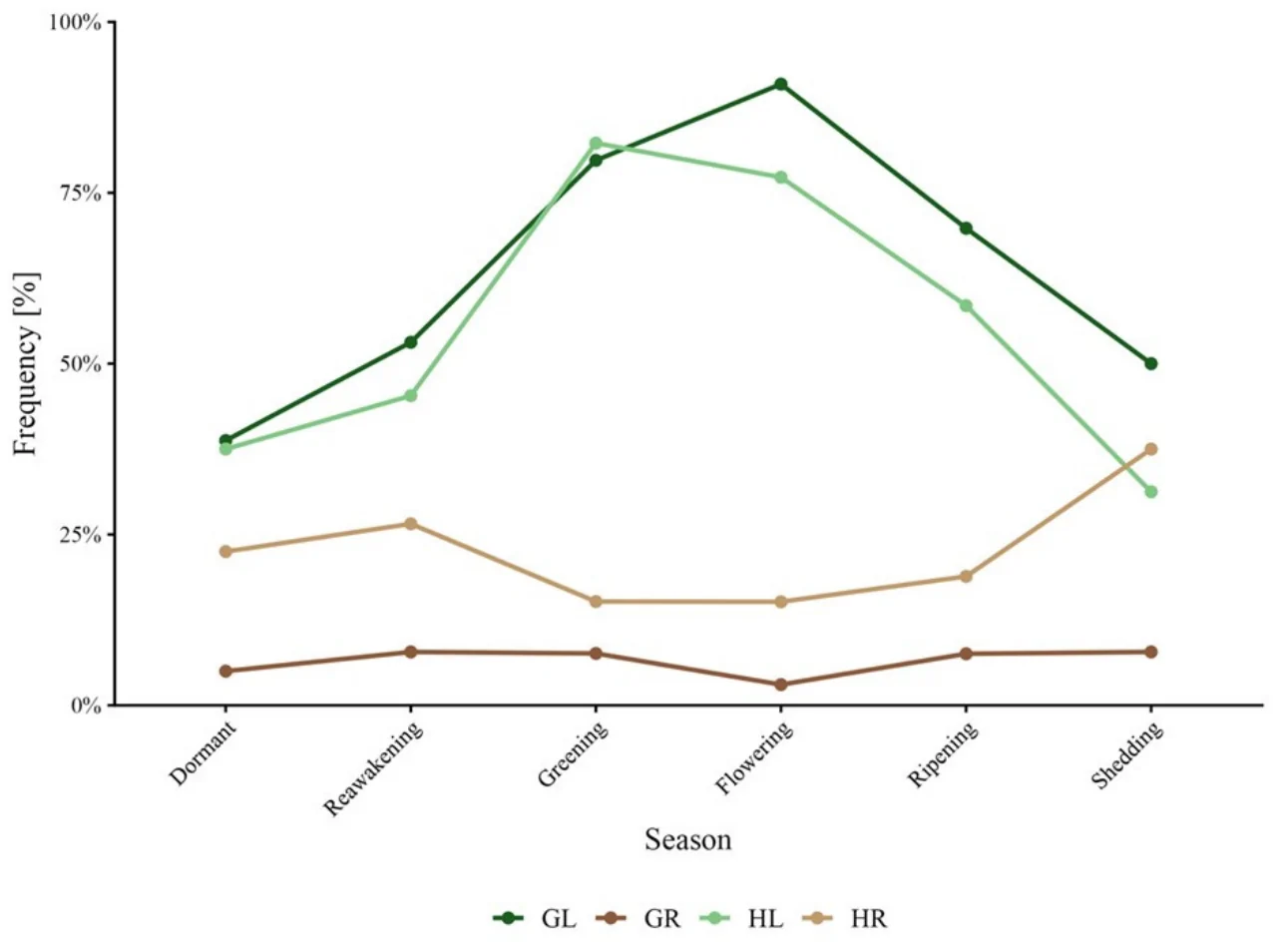
Dynamics of the frequency of occurrence of four understory plant fractions—grass leaves (GLs), grass roots (GRs), herb leaves (HLs), and herb roots (HRs)—in the stomach contents of 470 wild boars harvested in the Spała Forest District during the Dormant, Reawakening, Greening, Flowering, Ripening, and Shedding phenological seasons.

Seasonally, the frequency of the four fractions, GRs, HRs, GLs, and HLs, showed clear variation. In the case of GRs and HRs, seasonal dynamics were relatively limited, whereas markedly greater variability was observed for the aboveground components (GLs and HLs). In herbs, a distinct decrease in the frequency of roots was observed alongside a concurrent increase in the frequency of aboveground parts during the peak of the growing season. In contrast, in grasses, the frequency of roots remained relatively stable, while the frequency of aboveground parts exhibited pronounced seasonal dynamics, reaching a maximum during the Flowering period. For grasses, the frequency of GRs ranged from 3.0% to 7.8%, with the minimum (3.0%) recorded during Flowering, whereas in the remaining seasons the values were similar: Reawakening (7.8%), Greening (7.6%), Ripening (7.6%), and Shedding (7.8%). The frequency of this fraction did not differ significantly among seasons (Kruskal–Wallis χ^2^ = 2.3598, *p* > 0.05). Herb roots were recorded more frequently than grass roots, with frequencies ranging from 15.2% to 37.5% depending on the season, and these differences were statistically significant (Kruskal–Wallis χ^2^ = 19.7609, *p* < 0.05). The highest values were noted in the winter, autumn, and early spring seasons: Dormant (22.5%), Reawakening (26.6%), and Shedding (37.5%). The lowest frequencies of herb roots occurred during the peak growing seasons: Greening (15.2%), Flowering (15.2%), and Ripening (18.9%). Substantially greater seasonal dynamics were observed for aboveground parts in both grasses (Kruskal–Wallis χ^2^ = 63.2893, *p* < 0.05) and herbs (Kruskal–Wallis χ^2^ = 76.7896, *p* < 0.05). In the case of GLs, a systematic increase in frequency was observed, starting from Dormant (38.8%), through Reawakening (53.1%) and Greening (82.3%), and reaching a peak during Flowering (90.9%). In the subsequent two seasons, a marked decline in GL frequency was recorded: Ripening (69.8%) and Shedding, when GLs were present in 50% of the analyzed samples (Figure 2).

Significant differences were also found between the proportions of the GR, GL, HR, and HL fractions in the annual diet of wild boar, as well as among the six analyzed phenological periods. The mean values of the analyzed food fractions in the stomach contents of wild boar differed significantly among categories (Kruskal–Wallis χ^2^ = 342, df = 3, *p* < 0.05). The highest mean proportion was recorded for GLs–0.09 (±0.18), followed by HRs–0.07 (±0.21), HLs–0.04 (±0.13), and GRs–0.004 (±0.03). Post hoc analysis based on pairwise Wilcoxon tests with Holm correction revealed significant differences between all pairs of fractions. This indicates that the analyzed fractions were not utilized to a similar extent, and their proportions followed the sequence: GLs > HRs > HLs > GRs. Seasonal variation in the analyzed fractions was also pronounced and statistically significant (Kruskal–Wallis χ^2^ = 28.8, df = 5, *p* < 0.05). The combined proportion of the four analyzed fractions was highest during the Greening phase (0.33 ± 0.3), followed by Reawakening (0.29 ± 0.35), whereas the lowest value was recorded during the Ripening period (0.11 ± 0.2). Dunn’s post hoc tests with Holm correction revealed significant differences between the following seasons: Dormant and Greening, Greening and Ripening, and Greening and Shedding (*p* < 0.05).

**Figure 3:**
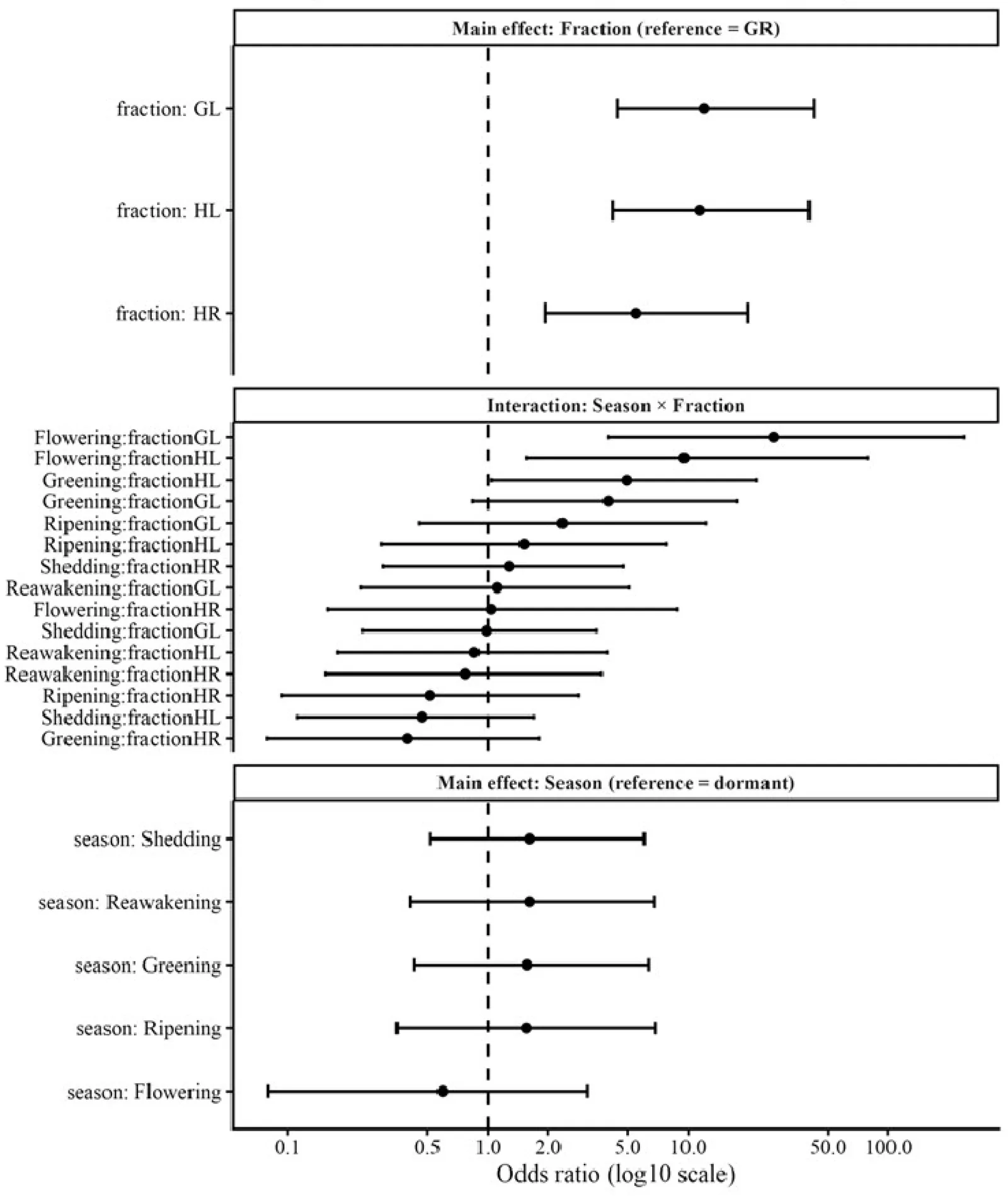
Effects of phenological season and plant fraction (GRs, GLs, HRs, and HLs) on the probability of occurrence in the stomach contents of 470 wild boars harvested in the Spała Forest District: odds ratios (ORs) with 95% confidence intervals (CIs) from the generalized linear model.

Results of the logistic regression model (GLM) for the presence of dietary fractions (presence~season × fraction) revealed a significant interaction between season and fraction type, indicating that the effect of season on occurrence probability depended on the type of food fraction. Compared with the reference fraction (GRs), the remaining fractions showed higher probabilities of occurrence. The odds ratios (ORs) for GLs, HLs, and HRs were mostly greater than 1, indicating a higher frequency of occurrence relative to GRs. In several cases, the confidence intervals did not include 1, suggesting significant differences from the reference fraction. A different pattern emerged for the interaction between season and fraction. Several combinations showed clear deviations from the reference level (GRs in the Dormant season), indicating that seasonal changes varied among fractions. The strongest positive interaction effects were observed for leaf fractions (GLs and HLs) during the Greening and Flowering seasons, where OR values were clearly above 1, indicating an increased probability of occurrence relative to the reference condition. In contrast, root fractions in the spring and summer seasons generally showed no clear increase in OR values, and in many cases their confidence intervals overlapped 1, suggesting no significance difference from the reference level. Notably, grass roots (GRs) during the Greening and Flowering periods tended to exhibit lower odds of occurrence compared with the Dormant season. In the Shedding season, an increase in the occurrence probability of root fractions was observed, particularly for HRs, indicating a seasonal shift toward belowground fractions in the later part of the year. For the main effect of season (reference = Dormant), no statistically significant effect was detected. Confidence intervals for individual seasons overlapped 1, indicating that, when averaged across all fractions, season alone did not significantly affect overall occurrence probability (Figure 3).

**Figure 4:**
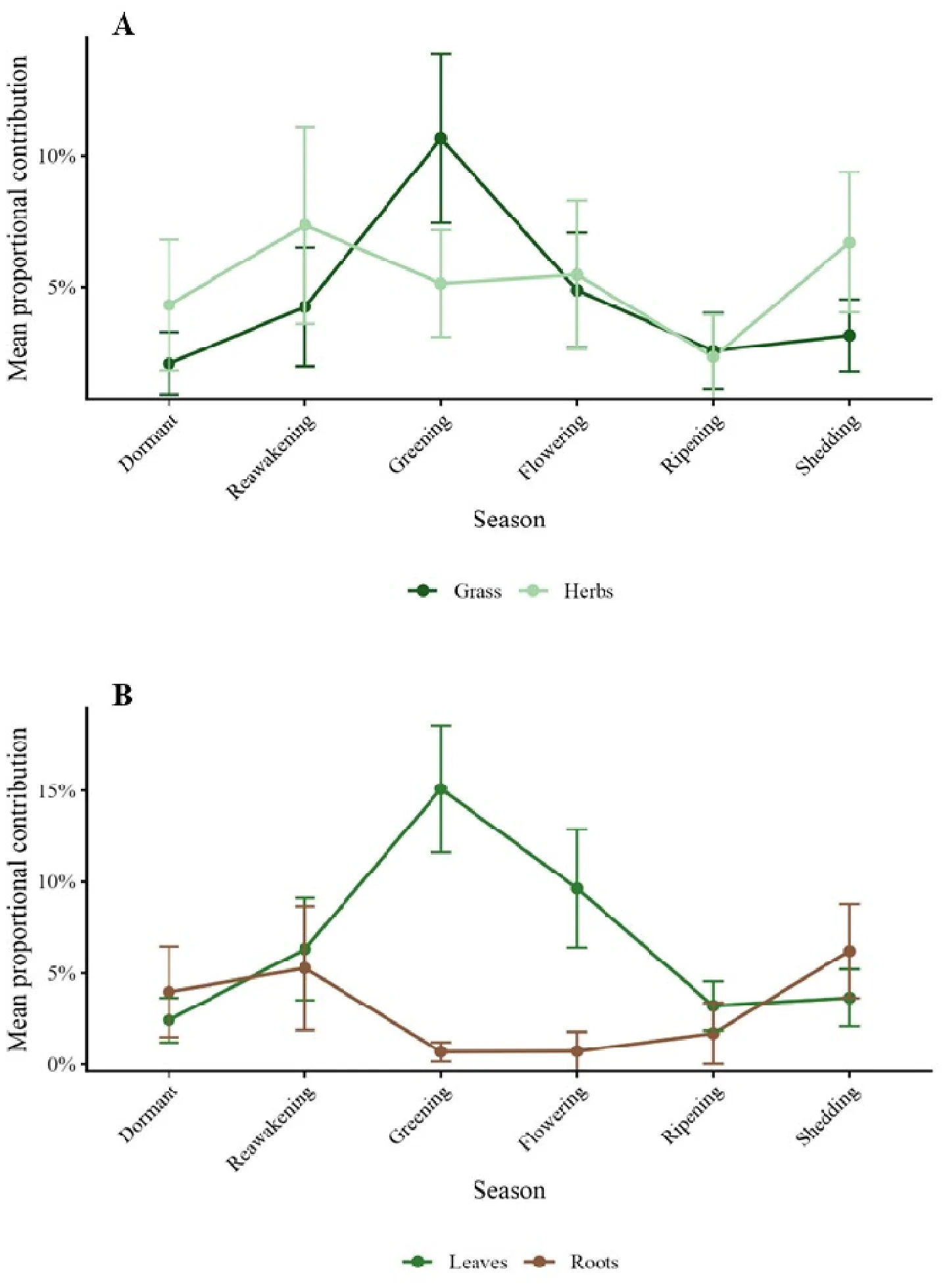
Seasonal dynamics of the proportions of (**A**) grasses and herbs and (**B**) aboveground and belowground plant parts in the stomach contents of 470 wild boars harvested in the Spała Forest District during six phenological seasons.

Comparison of the proportional shares of grasses and herbs in the wild boar diet, despite considerable seasonal variation, revealed no significant differences between these two food categories either throughout the year (Wilcoxon W = 429,627.0, *p* > 0.05) or across individual seasons (Wilcoxon W = 11,767, *p* > 0.05 for Dormant; Wilcoxon W = 7756.0, *p* > 0.05 for Reawakening; Wilcoxon W = 12,905.5, *p* > 0.05 for Greening; Wilcoxon W = 9097, *p* > 0.05 for Flowering; Wilcoxon W = 5724.5, *p* > 0.05 for Ripening; and Wilcoxon W = 30,990.0, *p* > 0.05 for Shedding). In contrast, significant seasonal variation was observed in the contribution of both food categories (χ^2^ = 67.7, df = 5, *p* < 0.001 for grasses; χ^2^ = 29.5, df = 5, *p* < 0.001 for herbs). In the case of herbs, after reaching a maximum during the Reawakening season (0.15 ± 0.29), their proportion gradually declined throughout the subsequent vegetation phases until Ripening, when the lowest share was recorded (0.05 ± 0.14). In contrast, grasses exhibited a clear increasing trend from the Dormant season (0.11 ± 0.04) to Greening, when the highest annual proportion was observed (0.21 ± 0.26), followed by a decline towards Shedding (0.06 ± 0.16) (Figure 4A). In contrast to the results obtained for grasses and herbs, analyses of the proportional shares of leaves and roots revealed significant differences between these food categories (Wilcoxon W = 618,789.5, *p* < 0.05), as well as pronounced seasonal variation in their contribution to the wild boar diet (χ^2^ = 102.3, df = 5, *p* < 0.05 for leaves; χ^2^ = 23.5, df = 5, *p* < 0.05 for roots). A distinct seasonal pattern was observed for leaves. Their proportion in the diet ranged from approximately 5% to over 30%, increasing from the Dormant season (0.05 ± 0.11) to a peak during Greening (0.30 ± 0.29) and subsequently declining markedly to similarly low levels during Ripening (0.06 ± 0.1) and Shedding (0.07 ± 0.18). The seasonal dynamics of the proportion of roots in the wild boar diet followed an opposing pattern, although it was less pronounced than that observed for aboveground plant parts. Roots accounted for approximately 1% to slightly more than 14% of the wild boar diet composition. The highest proportions were recorded during Shedding (0.12 ± 0.29), Reawakening (0.11 ± 0.27), and Dormant (0.08 ± 0.22). In contrast, the lowest shares, only slightly exceeding 1%, were observed during Greening (0.01 ± 0.05) and Flowering (0.01 ± 0.09). Differences between the mean proportions of leaves and roots proved significant despite similarities observed in some seasons. The proportion of roots relative to leaves was higher during Dormant (Wilcoxon W = 15,593.0, *p* < 0.05) and Shedding (Wilcoxon W = 37,539.5, *p* < 0.05), slightly lower during Reawakening (Wilcoxon W = 10,650.0, *p* < 0.05) and Ripening (Wilcoxon W = 8495.0, *p* < 0.05), and markedly lower during Greening (Wilcoxon W = 21,546.5, *p* < 0.05) and Flowering (Wilcoxon W = 15,435.0, *p* < 0.05), when leaves were consumed more than 20-fold more intensively during Greening and more than 10-fold more intensively during Flowering than roots (Figure 4B).

**Figure 5:**
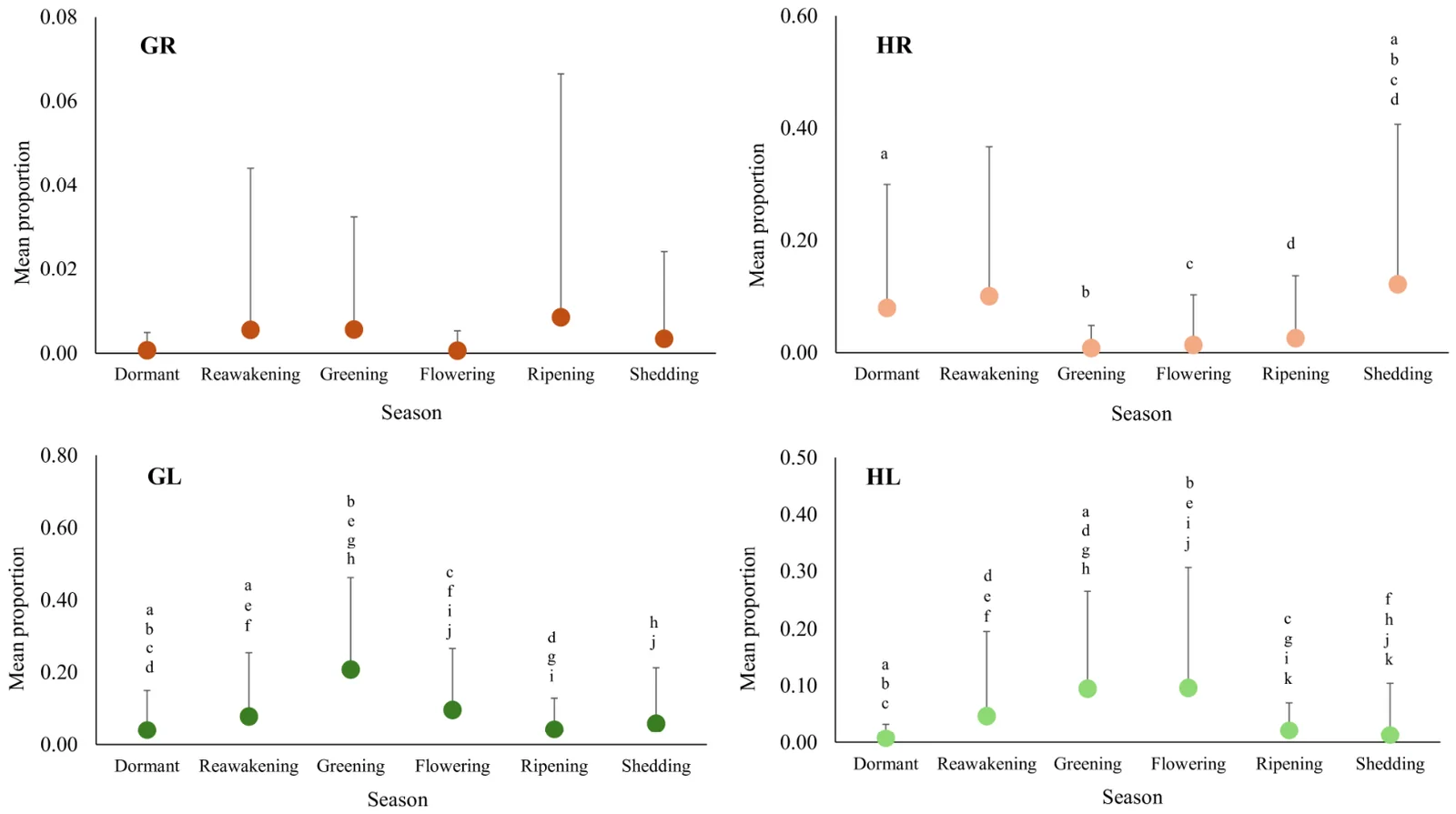
Comparison of the mean proportions of grass roots (GRs), grass leaves (GLs), herb roots (HRs), and herb leaves (HLs) in stomach contents of 470 wild boars harvested in the Spała Forest District among the Dormant, Reawakening, Greening, Flowering, Ripening, and Shedding phenological seasons (means sharing the same letter do not differ significantly at *p* < 0.05).

Seasonal differences were also identified when individual fractions were analyzed separately across seasons (Figure 5). In the case of GRs and HRs, significant differences were detected only for HRs (Wilcoxon W = 23.6558, *p* < 0.05). The highest proportional share of this fraction in the wild boar diet was recorded during Shedding (0.12 ± 0.29) and was significantly higher than during Dormant (0.08 ± 0.22; Wilcoxon W = 2.401, *p* < 0.05), Greening (0.01 ± 0.04; Wilcoxon W = 3.9424, *p* < 0.05), Flowering (0.01 ± 0.09; Wilcoxon W = 3.7564, *p* < 0.05), and Ripening (0.03 ± 0.11; Wilcoxon W = 2.9202, *p* < 0.05). Differences between the remaining seasons were not statistically significant (*p* > 0.05). No significant differences were found between the mean proportions of the GR fraction in the seasonal diet of wild boar (Wilcoxon W = 2.4653, *p* > 0.05). The proportion of this fraction ranged from approximately 1% during Dormant to approximately 9% during Ripening. Aboveground plant parts exhibited more pronounced seasonal dynamics, both in the case of GLs (Wilcoxon W = 71.251, *p* < 0.05) and HLs (Wilcoxon W = 96.7936, *p* < 0.05). In the case of GLs, their mean proportion in the analyzed samples ranged from approximately 1% to 21%. The highest values were recorded during Greening (0.21 ± 0.25) and were significantly higher than those observed during Dormant (0.04 ± 0.11; Wilcoxon W = 6.8076, *p* < 0.05), Reawakening (0.08 ± 0.17; Wilcoxon W = 4.2743, *p* < 0.05), Ripening (0.04 ± 0.09; Wilcoxon W = −3.4113, *p* < 0.05), and Shedding (0.01 ± 0.02; Wilcoxon W = −6.1131, *p* < 0.05). Flowering constituted the only exception, as the proportion of GLs was lower (0.10 ± 0.17) than during Greening, although this difference was not statistically significant (Wilcoxon W = −0.8493, *p* > 0.05). A similar seasonal pattern was observed for HLs. Their proportion throughout the year ranged from approximately 1% during Dormant (0.01 ± 0.03) to approximately 10% during Flowering (0.1 ± 0.17). The highest values were recorded during the summer seasons, namely Greening (0.10 ± 0.17) and Flowering (0.10 ± 0.21), between which no significant differences were detected (Wilcoxon W = −1.1821, *p* > 0.05). In contrast, the lowest proportions of HLs were observed during the autumn–winter seasons, Dormant (0.01 ± 0.03) and Shedding (0.01 ± 0.09), likewise without significant differences between them (Wilcoxon W = −0.6005, *p* > 0.05). The transitional seasons, Reawakening and Ripening, were characterized by intermediate and comparable proportions of HLs in the wild boar diet, amounting to 0.05 ± 0.15 and 0.02 ± 0.05, respectively (Wilcoxon W = 0.921, *p* > 0.05).

**Figure 6:**
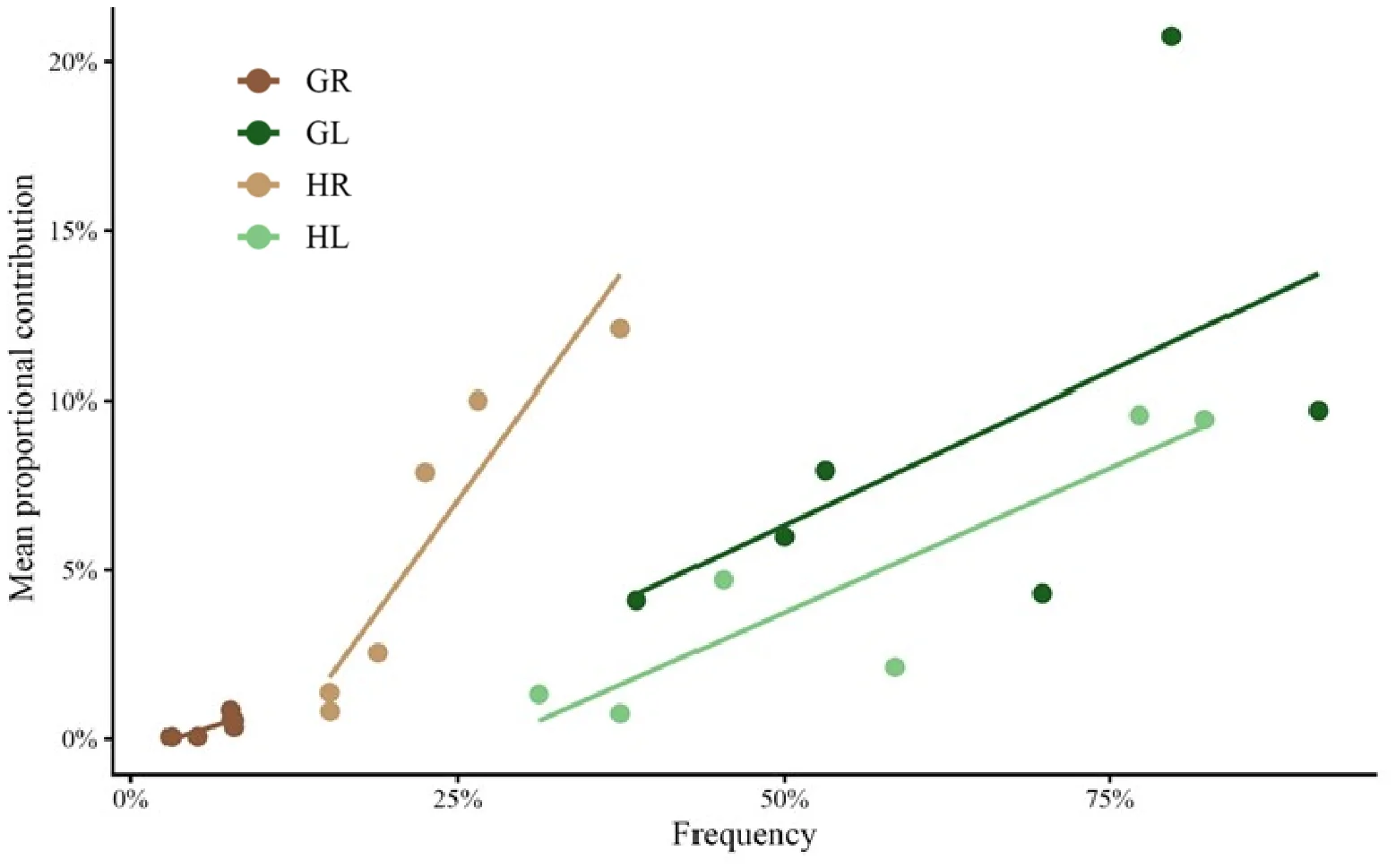
Relationships between the frequency of occurrence and mean proportions of grass roots (GRs), grass leaves (GLs), herb roots (HRs), and herb leaves (HLs) in the stomach contents of 470 wild boars harvested in the Spała Forest District.

The analysis of the relationship between occurrence frequency and the mean proportion of the analyzed dietary fractions in the wild boar diet revealed variation among individual components both in the strength of the relationships and in their statistical significance. However, in most cases no significant relationships were detected between the analyzed variables. The strongest associations were observed for the aboveground and belowground parts of herbs. In the case of HRs, a very strong and statistically significant correlation was found between occurrence frequency and the proportion of this fraction in the analyzed samples (Spearman r = 0.94; *p* < 0.05). The relationship between occurrence frequency and the proportion of HLs in the analyzed samples remained at the threshold of statistical significance (Spearman r = 0.83; *p* > 0.05). In contrast, no significant relationships were detected for the aboveground and belowground parts of grasses (GRs: Spearman r = 0.46, *p* > 0.05; GLs: Spearman r = 0.77, *p* > 0.05) (Figure 6).

**Figure 7:**
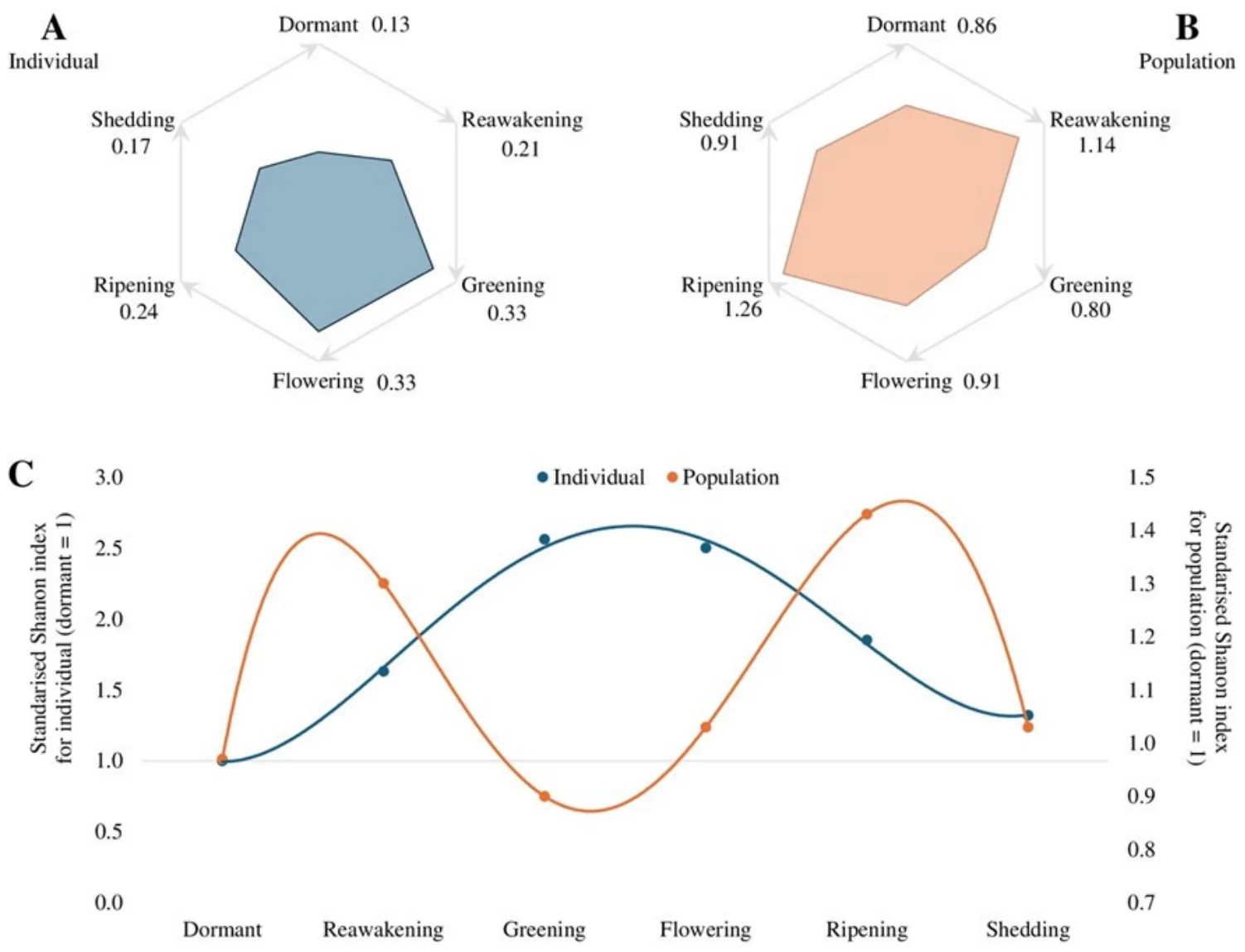
Seasonal variation in the Shannon diversity index (H′) of dietary fractions (GRs, GLs, HRs, and HLs) in the stomach contents of 470 wild boars harvested in the Spała Forest District. (**A**) Mean individual dietary diversity calculated for each sample and averaged across seasons; (**B**) population-level dietary diversity calculated from mean seasonal proportions of dietary fractions; (**C**) comparison of relative Shannon index values between the individual- and population-level approaches, standardized to the Dormant season (=1).

Shannon diversity index values, calculated independently for individual wild boars and for the entire population across successive seasons, revealed clear differences in the diversity of the four analyzed dietary fractions among phenological phases (Figure 7). In the individual-level analysis (Figure 7A), in which the H′ index was calculated for individual samples and subsequently averaged within seasons, the lowest level of diversity was recorded during Dormant (H′ = 0.127). An increase in diversity was subsequently observed during Reawakening (H′ = 0.127), whereas the highest values were recorded during Greening and Flowering (H′ = 0.333 and H′ = 0.325, respectively). During the subsequent seasons, Ripening and Shedding, diversity decreased again (H′ = 0.241 and H′ = 0.171, respectively). The low H′ values (<0.4) resulted from the limited number of analyzed dietary fractions and the high proportion of samples containing only one or two of them. In the population-level analysis (Figure 7B), based on the mean proportions of dietary fractions across all wild boars obtained in a given season, higher H′ values were obtained than in the individual-level analysis. The lowest values were recorded during Greening (H′ = 0.803) and Dormant (H′ = 0.857), whereas the highest occurred during Reawakening (H′ = 1.142) and Ripening (H′ = 1.255). Intermediate values were observed during the remaining seasons (Flowering: H′ = 0.908; Shedding: H′ = 0.913). Comparison of the relative Shannon diversity index values between the individual- and population-level approaches, following standardization relative to the Dormant season (value = 1) (Figure 7C), revealed distinct patterns of seasonal variation in dietary diversity. In the individual-level analysis, the highest relative values were observed during Greening and Flowering, when all wild boars utilized the four analyzed dietary fractions in relatively similar proportions, resulting in the lowest variability in their contribution at the population level. In contrast, the population-level analysis showed maximum values during Ripening and Reawakening, which coincided with the lowest diversity values at the individual level. This indicates the dominance of a single dietary fraction—often differing among individuals—was accompanied by a low contribution of the remaining fractions, which were likewise variable between individuals.

**Figure 8:**
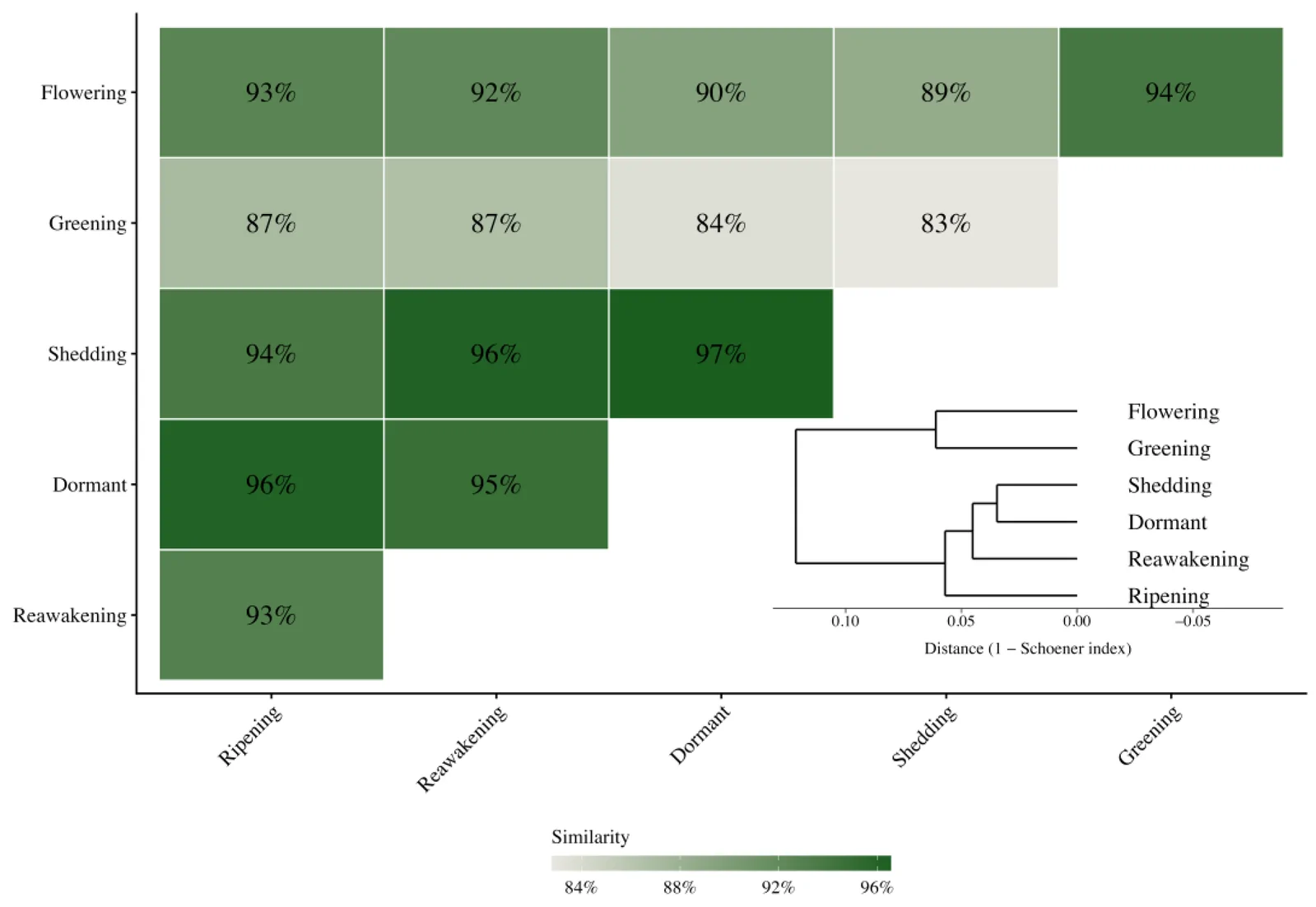
Similarity in the composition of grass roots (GRs), grass leaves (GLs), herb roots (HRs), and herb leaves (HLs) among the Dormant, Reawakening, Greening, Flowering, Ripening, and Shedding phenological seasons, based on Schoener’s index calculated from the stomach contents of 470 wild boars harvested in the Spała Forest District.

The similarity in the composition of the GR, GL, HR, and HL fractions among seasons was generally high and ranged within a relatively narrow interval of Schoener’s index values (83–97%). The highest similarity values (97%) were recorded between the autumn–winter seasons Dormant and Shedding. Slightly lower values (96%) were observed for the pairs Shedding–Reawakening and Dormant–Ripening. The lowest similarity occurred between Dormant and Greening (84%) and between Greening and Shedding (83%). In the remaining cases, seasonal similarity ranged from 89% to 94%. The arrangement of seasons on the dendrogram confirmed these relationships, indicating clustering of phenological phases according to trophic similarity. The spring–summer seasons (Greening, Flowering) formed a distinct group, and the autumn–winter seasons (Shedding, Dormant) were the most like each other, whereas the transitional phases (Reawakening, Ripening) occupied intermediate positions (Figure 8).

## 4. Discussion

Morphological and behavioral adaptations for intensive rooting and the efficient extraction of food from beneath the soil surface and its upper layers enable wild boar to exploit understory vegetation throughout the year [33,41,56]. Grasses and herbs appear to provide a stable, year-round food resource, with wild boar exploiting both their belowground and aboveground parts, although to varying degrees. Despite pronounced seasonal shifts in the contribution of individual dietary fractions, the overall contribution of grasses and herbs remained remarkably consistent among successive phenological seasons, with similarity exceeding 80%. This high degree of seasonal consistency suggests that the forest understory represents a permanent foraging habitat for wild boars, whose diet, despite their omnivorous feeding habits, is based predominantly on plant-derived resources [5,41,57].

Although seasonal differences in the use of grasses and herbs by wild boar were characterized by considerable variability, they nevertheless revealed clear seasonal patterns. First, grasses exhibited substantially greater seasonal variation than herbs. Their lowest contribution was recorded during winter (Dormant) and again in late summer and autumn (Ripening and Shedding), whereas their highest contribution, approximately ten-fold greater, occurred during the period of intensive spring vegetation growth (Greening). In contrast, herbs showed less pronounced seasonal variation. Their greatest contribution to the analyzed samples was recorded in early spring (Reawakening), before grasses reached their peak contribution, and again in autumn (Shedding), which may reflect the attractiveness of their roots as a food resource.

The increased use of herbs in autumn may also be associated with the depletion of food resources in agricultural fields following harvest. As food resources become progressively depleted within agricultural–forest mosaics, wild boars shift their foraging activity towards forest understory vegetation [58,59]. Within this vegetation layer, herbs, particularly their roots, represent a food resource that is actively sought and readily consumed by wild boar [1,8]. Belowground organs of herbaceous plants, including roots, tubers and bulbs, constitute a widespread and important component of the wild boar diet throughout the species’ distribution range. In general, wild boars preferentially consume fleshy roots and belowground storage organs rather than woody roots [60]. These resources may become particularly important during autumn and winter [1,24].

Seasonal changes in the contribution of the four dietary fractions closely tracked the phenological development of vegetation, suggesting that wild boar may flexibly adjust their foraging strategy to seasonal changes in plant resources. Such behavioral flexibility is characteristic of animals inhabiting strongly seasonal environments [59,61]. In our study, the highest contribution of aboveground plant parts, namely grass and forb leaves, occurred during the Greening and Flowering phases, corresponding to the period of rapid aboveground biomass production. During this period, young plant tissues are rich in protein, highly digestible, and only weakly lignified, making them particularly attractive food resources [62].

A contrasting pattern emerged in the use of grasses and herbs between the two consecutive phenological phases representing spring and summer. Whereas the occurrence of grass leaves increased markedly from the Greening to the Flowering phase, the importance of forb leaves declined slightly over the same period, marking the beginning of a progressive decrease that continued until the Dormant phase. This pattern may indicate a shift in the relative attractiveness of the two plant groups as the growing season progresses, or changes in the relationship between their availability and nutritional value.

At the same time, belowground organs of both grasses and herbs constituted only a minor component of the diet during the Greening and Flowering phases. In the case of forb roots, their frequency of occurrence declined by approximately one-third between winter (Dormant) and spring (Greening), whereas their proportion in the diet decreased nearly ten-fold. The opposite pattern was observed during autumn and winter. Between the Ripening and Shedding phases, the frequency of forb roots approximately doubled, while their dietary proportion increased almost five-fold. This pattern most likely reflects the declining nutritional quality of aboveground plant tissues towards the end of the growing season together with the high concentration of stored reserves in the belowground storage organs of many forb species [63]. In contrast, grass roots remained a consistently minor dietary component, both in terms of occurrence and proportional contribution, showing little seasonal variation.

The observed differences between grasses and herbs suggest that seasonal changes in their contribution to the wild boar diet are driven not only by plant phenology but also by differences in the ecological and nutritional characteristics of the two plant groups. The observed patterns can be interpreted in the context of at least two complementary, rather than mutually exclusive, mechanisms.

The first mechanism is likely related to the greater abundance of grasses than herbs within the study area, largely resulting from the extensive occurrence of managed grasslands, where grasses are the dominant vegetation. Owing to their management, particularly spring mowing (the first cut), the high nutritional quality of grasses is maintained longer than that of herbs and persists until the Flowering phase, a pattern reflected in the composition of wild boar stomach contents. In contrast, most herbs occur outside agricultural areas, where their growth typically peaks during spring. As the growing season progresses, tissue lignification reduces their nutritional quality as forage for wild boar. Consequently, their occurrence in the diet reaches its maximum during the Greening phase and then gradually declines.

A second, equally plausible mechanism explaining the contrasting seasonal dynamics in the consumption of aboveground and belowground plant parts is the difference in their nutritional value. Herb leaves generally have slightly higher nutritional value than grass leaves. In contrast, the belowground storage organs of herbs, including tubers, bulbs, rhizomes and enlarged roots, contain substantially higher concentrations of readily available reserve compounds than the belowground organs of grasses [64,65,66,67]. These organs are also characterized by high digestibility and energy content [68].

During the period of rapid vegetation growth, when plant biomass is abundant, the energetic benefits of selectively searching for the most nutritious plant species may not outweigh the additional costs associated with locating them. Under such conditions, opportunistic exploitation of readily available resources, particularly grasses, is likely to be the more profitable strategy. The opposite situation occurs during autumn and winter, when food becomes less abundant. Under these conditions, the nutritional quality of individual food resources becomes increasingly important, and the energetic benefits of locating and excavating the belowground storage organs of herbs may outweigh the additional costs of obtaining them. Consequently, the increased contribution of belowground herb organs to the autumn diet of wild boar may reflect a seasonal shift in foraging strategy aimed at maximizing net energetic gain.

Wild boars are widely regarded as opportunistic generalist feeders [5]. Their broad trophic niche, together with well-documented seasonal, annual and regional variation in diet composition, indicates that food-resource use is determined primarily by resource availability [41]. Our findings on the use of understory vegetation do not challenge this view but suggest that opportunistic foraging in wild boars is not a fixed strategy. During periods of food abundance, wild boars are likely to forage more opportunistically, concentrating primarily on the most readily accessible resources. In contrast, when food becomes scarce, they appear to become more selective, favoring resources that provide the greatest net energetic return, even when locating and acquiring them requires greater expenditure of time and energy. Such seasonal shifts in foraging strategy are consistent with the predictions of optimal foraging theory, which proposes that animals maximize the balance between the energetic value of food and the costs of obtaining it [69,70]. In light of our findings, this suggests that seasonal variation may influence foraging decisions in wild boar through changes in the availability and potential energetic value of different food resources.

Another noteworthy finding, which has received little attention in previous studies of wild boar diet, is the difference between patterns of dietary diversity observed at the individual and population levels. During periods of limited food availability, particularly in autumn, winter and early spring, the diet of individual wild boars was relatively simple and often dominated by a single dietary fraction. At the same time, especially during the Reawakening phase and the post-harvest period (Ripening), different individuals relied on different dietary fractions, resulting in greater dietary diversity at the population level. This pattern may reflect both the opportunistic foraging behavior of wild boars and the pronounced spatial heterogeneity of forest understory resources [5,9,71]. Under conditions of limited food availability, individual wild boars may concentrate on those resources that not only provide high energetic returns but are also the most readily available within their immediate surroundings.

The opposite pattern was observed during the period of rapid vegetation growth. At that time, all four dietary fractions were widely available, allowing individual wild boars to consume a more diverse diet. At the population level, however, most individuals exploited a similar set of food resources, resulting in a relative homogenization of diet among individuals. These findings indicate that seasonal variation in resource availability influences not only diet composition but also the degree of individualization in foraging strategies within the population.

Although it remains unclear to what extent the high seasonal similarity observed among the four dietary fractions may be attributable to contemporary climate change, this possibility cannot be excluded. This hypothesis, however, requires direct testing. Nevertheless, the increasing frequency of mild winters with little or no snow cover and limited soil freezing is likely to increase the year-round accessibility of the belowground organs of understory plants. These conditions may facilitate more intensive rooting in the upper soil layers, thereby increasing the use of understory vegetation, including both leaves and roots [72,73]. At the same time, above-freezing winter temperatures allow many grass species to remain vegetatively active, providing a source of fresh biomass outside the traditional growing season [74]. Under such conditions, wild boars may exploit food resources that were previously seasonally unavailable or substantially less accessible.

Our findings therefore suggest that one potential consequence of contemporary climate change may be an increased importance of forest understory vegetation as a year-round food resource for wild boar. It should be emphasized, however, that the understory vegetation examined in this study represents only one component of the much broader foraging environment available to this species.

In conclusion, our findings demonstrate that wild boars exploit understory vegetation throughout the year. They exhibit a high degree of trophic plasticity, seasonally adjusting their use of understory vegetation in response to plant phenology and the changing availability of food resources. Despite pronounced seasonal shifts in the relative importance of individual dietary fractions, understory vegetation remains a persistent, year-round component of the wild boar trophic niche. At the same time, patterns of resource use differ between the individual and population levels, highlighting both the complexity of wild boar foraging strategies and their remarkable capacity to adapt to changing environmental conditions. Taken together, these findings suggest that the remarkable ecological adaptability of wild boar is associated not only with a broad trophic niche but also with the ability to seasonally adjust foraging decisions in response to changes in resource availability and the energetic profitability of food resources within the understory.

## 5. Conclusions

1. Wild boars exhibit pronounced seasonal dietary selectivity within understory vegetation, adjusting their use of aboveground and belowground plant parts in response to plant phenology and the seasonal availability of food resources. During periods of intensive vegetation growth, grass leaves constituted the dominant dietary component, whereas the importance of herbaceous plant roots increased during autumn and winter.
2. The strongest seasonal shifts were observed in aboveground plant parts, whose contribution to the wild boar diet increased markedly during spring and summer. In contrast, belowground fractions generally showed lower seasonal variability, although the contribution of herb roots increased during periods when aboveground plant resources became less prominent in the diet.
3. Seasonal variation in dietary diversity was associated with changes in the occurrence and contribution of individual dietary fractions and with inter-individual differences in foraging strategies. Individual diets showed greater specialization during periods when fewer dietary fractions were recorded, whereas greater diversity of exploited dietary fractions was observed at the population level.
4. Despite seasonal variation in the contribution of individual dietary fractions, dietary similarity among seasons remained high. This finding indicates that understory vegetation and the upper soil layers constitute a stable, year-round component of the wild boar trophic niche, the importance of which may potentially increase under conditions of mild winters and ongoing climate change.

## Figures and Tables

**Table 1:** Distribution of samples by number of fractions (values in parentheses: frequency of specific fraction combinations: GRs—grass roots, GLs—grass leaves, HRs—herbs roots, HLs—herbs leaves).

Season	Number of Samples with a Given Number of Fractions (in Parentheses: Numbers of Specific Combinations)					Total
	4	3	2	1	0	
Dormant	2	2 (HRs × GLs × HLs–2)	22 (GLs × HLs–10 HLs × HRs–8 HRs × GLs–2 GRs × HRs–1 GRs × HLs–1)	25 (GLs–15 HLs–7 HRs–3)	29	80
Reawakening	0	5 (HRs × GLs × HLs–4 GRs × HRs × GLs–1)	24 (GLs × HLs–15 HRs × HLs–4 HRs × GLs–2 GRs × HLs–1 HRs × HLs–1 GRs × GLs–1)	22 (GLs–11 HRs–5 HLs–4 GRs–2)	13	64
Greening	2	10 (HRs × GLs × HLs–7 GRs × GLs × HLs–3)	45 (GLs × HLs–43 GRs × GLs–1 HRs × GLs–1)	18 (HLs–10 GLs–6 HRs–2)	4	79
Flowering	0	10 (HRs × GLs × HLs–9 GRs × GLs × HLs–1)	39 (GLs × HLs–37 HLs × GRs–1 HRs × GLs–1)	15 (GLs–12 HLs–3)	2	66
Ripening	1	7 (HRs × GLs × HLs–6 GRs × GLs × HLs–1)	17 (GLs × HLs–14 HRs × HLs–1 GRs × HLs–1 HRs × GLs–1)	23 (GLs–14 HLs–7 HRs–1 GRs–1)	5	53
Shedding	0	18 (HRs × GLs × HLs–13 GRs × HRs × GLs–2 GRs × GLs × HLs–2 GRs × HRs × HLs–1)	27 (GLs × HLs–10 HRs × HLs–8 HRs × GLs–6 GRs × GLs–2 GRs × HRs–1)	54 (GLs–29 HRs–17 HLs–6 GRs–2)	29	128
Total	5 (1.1%)	52 (11.1%)	174 (37.0%)	157 (33.4%)	82 (17.4%)	470 (100%)

## Data Availability

The raw data supporting the conclusions of this article will be made available by the authors upon request.
